# The Involvement of Apoptosis Inhibitor of Macrophage in the Disease Severity of Primary Biliary Cholangitis

**DOI:** 10.3390/jcm15031169

**Published:** 2026-02-02

**Authors:** Takashi Himoto, Erika Mori, Manami Tanimoto, Koji Fujita, Shima Mimura, Tomoko Tadokoro, Kyoko Oura, Joji Tani, Asahiro Morishita, Hideki Kobara

**Affiliations:** 1 Department of Medical Technology, Kagawa Prefectural University of Health Sciences, Takamatsu 761-0123, Kagawa, Japan; 2 Department of Gastroenterology and Neurology, Faculty of Medicine, Kagawa University, Takamatsu 761-0793, Kagawa, Japan; fujita.koji@kagawa-u.ac.jp (K.F.); mimura.shima@kagawa-u.ac.jp (S.M.); tadokoro.tomoko@kagawa-u.ac.jp (T.T.); oura.kyoko@kagawa-u.ac.jp (K.O.); tani.joji.kb@kagawa-u.ac.jp (J.T.); morishita.asahiro@kagawa-u.ac.jp (A.M.); kobara.hideki@kagawa-u.ac.jp (H.K.)

**Keywords:** apoptosis inhibitor of macrophage, obesity, insulin resistance, type 2 diabetes mellitus, disease severity, primary biliary cholangitis

## Abstract

**Background**: A protein called ‘apoptosis inhibitor of macrophage (AIM)’ is involved in the pathogenesis of obesity-associated disease. Although it is widely recognized that concurrent obesity affects the disease progression of chronic liver disease, as does concurrent type 2 diabetes mellitus (T2DM), the involvement of AIM in the pathogenesis of obesity or insulin resistance is not yet understood in patients with primary biliary cholangitis (PBC). **Methods**: Obesity was defined as a body mass index (BMI) exceeding 25, and insulin resistance was defined as a homeostasis model assessment for insulin resistance (HOMA-IR) value exceeding 2.0, respectively. Hepatic steatosis was estimated based on the classification proposed by Brunt and colleagues. The histological stage was determined by Scheuer’s classification. **Results**: Twelve (25.0%) of the forty-eight PBC patients had concurrent obesity, and seven (14.6%) had concurrent T2DM. The PBC patients with obesity had significantly higher frequency of hepatic steatosis. Compared to the patients without T2DM, those with concurrent T2DM had significantly higher serum ALT levels and more advanced histological stages. The patients’ serum AIM levels were not associated with concurrent obesity or concurrent T2DM. Our analyses identified the following as the factors that significantly affected the patients’ AIM levels: serum immunoglobulin G, albumin, tumor necrosis factor-α levels, and the histological stages. **Conclusions**: These results indicate that AIM may not be involved in obesity or insulin resistance, but it may be associated with the disease severity of PBC.

## 1. Introduction

Obesity affects the progression of chronic liver diseases (CLDs), including metabolic dysfunction-associated steatotic liver disease (MASLD) and hepatitis C virus (HCV)-related CLD [1,2,3,4]. In patients with MASLD or HCV-related CLD, obesity is associated with advanced liver steatosis and fibrosis. The involvement of obesity in the progression of primary biliary cholangitis (PBC) has been confirmed [5,6]. PBC is a chronic cholestatic disease characterized by the progressive destruction of intrahepatic bile ducts via an autoimmune response [7]. Concurrent type 2 diabetes mellitus (T2DM) has also been shown to be associated with advanced liver fibrosis in those patients with CLDs [1,8,9]. However, the prevalence of concurrent T2DM in patients with PBC is not yet known.

Obesity and overweight are also associated with autoimmune diseases, including systemic lupus erythematosus (SLE) and rheumatoid arthritis (RA). Obesity eventually contributes to more severe inflammation in patients with such autoimmune diseases. Several putative mechanisms underlying this facilitation of inflammation in autoimmune diseases have been proposed. Several types of adipokines, the protein known as “apoptosis inhibitor of macrophage” (AIM), Th17 cells, alteration of gut microbiota, and vitamin D deficiency may play crucial roles in the progression of autoimmune diseases [10,11]. Insulin resistance is also associated with more advanced disease activity among individuals with autoimmune diseases [12,13].

AIM is a member of the family of scavenger receptors and is identified as an apoptosis inhibitor that supports the survival of macrophages [14]. An increase in the serum AIM level may cause lipolysis under the condition of obesity, leading to the release of a large amount of saturated free acids from adipocytes. This release results in the facilitation of chemokine production in adipocytes and a subsequent migration of M1 macrophages. The recruitment of M1 macrophages into adipose tissues is likely to evoke systemic inflammation and finally lead to insulin resistance [15]. It has been demonstrated that AIM is ultimately involved in the process of hepatocarcinogenesis in patients with MASLD [16].

AIM is associated with the molecule IgM pentamer, and it retains an IgM/autoantigen complex on the surface of follicular dendritic cells by interfering with the binding of the complex to the FCα/μ receptor. This reaction results in the presentation of an IgM-dependent autoantigen to germinal-center-B cells, leading to the production of IgG autoantibody. These processes imply that AIM may be responsible for the obesity-associated autoimmune response [17].

The primary purpose of this study was to determine whether concurrent obesity and/or T2DM potentially affect the disease severity in PBC patients. Moreover, we also investigated whether AIM might be involved in the pathogenesis of obesity-associated autoimmunity in PBC patients with concurrent obesity or T2DM.

## 2. Patients and Methods

### 2.1. Study Population

This was a single-center retrospective observational study. Forty-eight patients with PBC, who were admitted to the Hospital of Kagawa University Faculty of Medicine between 2005 and 2015, and were histologically confirmed, were assigned in this study. Liver specimens were missing in 3 patients among the 48 patients with PBC. Therefore, we did not perform the histological evaluation in these three patients. The patients’ clinical diagnoses of PBC were based on the internationally accepted criteria [18]. As comparison groups, eleven patients with autoimmune hepatitis (AIH) and eight cases of healthy control (HC) were assigned in this study. AIH was diagnosed based on the revised scoring system proposed by the International Autoimmune Hepatitis Group [19]. Type 2 diabetes mellitus (T2DM) was diagnosed in accordance with the international criteria [20]. Obesity was defined in this study as a body mass index (BMI) exceeding 25 [21], because this definition is widely accepted in Japan.

The study protocol complied with all of the provisions of the Declaration of Helsinki. The design of this study was approved by the Ethical Committees of the Kagawa Prefectural University of Health Sciences (#212). The requirement for patients’ informed consent was waived due to the study’s retrospective design and use of anonymized patients’ data.

### 2.2. Laboratory Assessments

Serum aminotransferase (ALT), alkaline phosphatase (ALP), total bilirubin (T-Bil), albumin (Alb), total cholesterol (T-Cho), triglycerides (TG), immunoglobulin M (IgM), and immunoglobulin G (IgG) levels, in addition to hemoglobin A1c (HbA1c), were measured using standard routine laboratory techniques in the PBC, AIH, and HC groups. Insulin resistance was estimated based on the homeostasis model for the assessment of the insulin resistance (HOMA-IR) value, using the following equation: HOMA-IR value = fasting insulin (μU/mL) × fasting glucose (mg/dL)/405. Insulin resistance was defined as a HOMA-IR value exceeding 2.0.

Commercially available enzyme-linked immunosorbent assay (ELISA) kits were used for the measurement of antimitochondrial antibodies (MESA CUP-2 Test Mitochondrial M2 kit, Medical & Biological Laboratories, Nagoya, Japan), anti-gp210 antibodies (Inova Diagnostics, San Diego, CA, USA), and anti-Sp100 antibodies (Inova Diagnostics, San Diego, CA, USA). Anti-centromere antibodies (ACAs) were defined as a discrete speckled pattern on HEp-2 cells by an indirect immunofluorescent method with 1:40 or more dilution. AIM (Medical & Biological Laboratories) and TNF-α (Inova Diagnostics) levels were also determined by commercially available ELISA kits.

### 2.3. Histological Assessments

Liver specimens were obtained by liver biopsy from each PBC and AIH patient under ultrasound guidance before treatment. The number of portal tract in each liver specimen was sufficient to evaluate histological findings. The pathologist was blinded to the clinical information on the enrolled patients. The tissue samples were fixed in 10% formalin and embedded in paraffin. The tissue sections were stained with hematoxylin and eosin for pathological evaluation. The histological stage of PBC was identified based on Scheuer’s classification [22]. Scheuer’s stage 1 to stage 4 were scored as 1–4 points, respectively. The grade of hepatic steatosis was estimated based on the classification proposed by Brunt and colleagues [23].

### 2.4. Statistical Analyses

The results of the statistical analyses are presented as means ± standard deviations (SDs). The Mann–Whitney *U*-test was used for the comparisons of variables between two groups, and the Bonferroni/Dunn method was used for the comparison of three groups. Fisher’s exact probability test was used to compare the difference in frequencies. The relationships between quantitative variables were analyzed by Pearson’s test. Multivariate analysis was performed using logistic regression. Probability (*p*)-values less than 0.05 were considered significant.

## 3. Results

### 3.1. The Clinical Characteristics of the PBC Patients

Table 1 summarizes the clinical characteristics of the 48 patients with PBC. Concurrent obesity was observed in 12 (25.0%) of the PBC patients, and concurrent T2DM was observed in seven (14.6%) of the patients. Six (24.0%) of the 25 patients with PBC whose HOMA-IR values were available had HOMA-IR values exceeding 2.0. Five (11.1%) of the forty-five patients had grade 1 hepatic steatosis, while none of the PBC patients had grade 2 or grade 3 hepatic steatosis.

### 3.2. Comparison of the Serum AIM Level in Each Group

**Figure 1:**
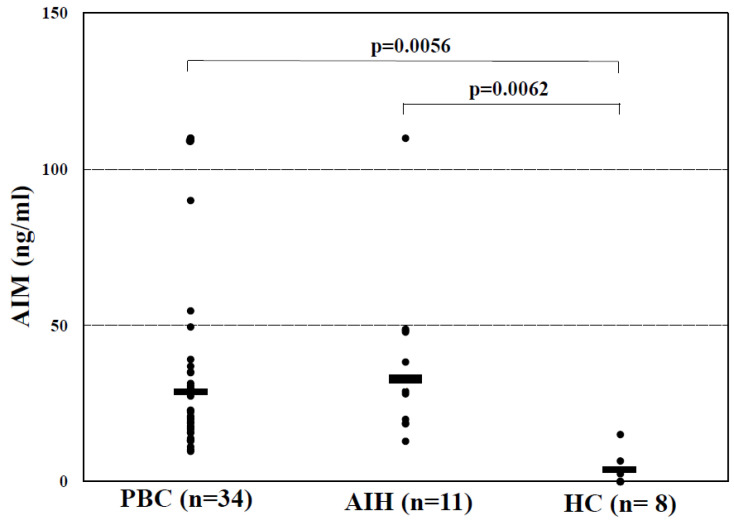
Serum AIM levels in the groups of PBC, AIH, and HC. The horizontal bar represents the mean AIM level in each group.

As shown in Figure 1, serum AIM levels were significantly higher in the PBC patients, compared to those in the HCs (33.1 ± 28.9 vs. 3.0 ± 5.4 ng/mL, *p* = 0.0056). The serum AIM level of the patients with AIH were approximately equivalent to those of the patients with PBC, and were also significantly higher than those of the HCs (38.2 ± 27.2 vs. 3.0 ± 5.4 ng/mL, *p* = 0.0062).

### 3.3. The Clinical Characteristics of the PBC Patients with Concurrent Obesity

We compared the laboratory and histological data between the PBC patients with and without obesity. As shown in Table 2, no significant differences in serum T-Cho and TG levels and HbA1c were found between the obese and non-obese PBC patients, although PBC patients with obesity had significantly higher BMIs. Regarding the liver function test, serum ALP levels were significantly lower in the PBC patients with concurrent obesity compared to those in the PBC patients without obesity (383 ± 126 vs. 650 ± 383 IU/L, *p* = 0.0172). However, there was no significant difference in the serum ALT, Alb, and T-Bil levels between the obese and non-obese PBC patients. Likewise, the PBC patients with and without obesity also showed no significant differences in the titers of anti-M2, anti-gp210, and anti-Sp100, as well as the positive rate of ACAs.

The histological stages were also approximately equivalent between these two groups. However, the frequency of concurrent hepatic steatosis was significantly higher in PBC patients with obesity than that of PBC patients without obesity (40.0% vs. 2.9%, *p* = 0.0062).

### 3.4. The Clinical Characteristics of the PBC Patients with Concurrent T2DM

Table 3 presented the laboratory and histological findings for the PBC patients with and without concurrent T2DM. There was no significant difference in BMI, serum T-Cho, and TG levels between PBC patients with and without T2DM, although PBC patients with T2DM had significantly higher HbA1c. The PBC patients with T2DM had significantly higher serum ALT levels (68 ± 24 vs. 47 ± 27 IU/L, *p* = 0.0486). However, serum T-Bil and Alb levels were roughly the same between the PBC patients with and without T2DM. Regarding histological findings, PBC patients with concurrent T2DM had significantly more severe histological stages (2.8 ± 1.0 vs. 1.8 ± 0.9, *p* = 0.0313), compared to those without T2DM, indicating that PBC patients with concurrent T2DM had more advanced stages. There was no significant difference in the frequency of hepatic steatosis between the groups. No significant differences in the titers of anti-M2, anti-gp210, and anti-Sp100 were observed between these groups. The serum AIM, IgM, and IgG levels were not dependent on concurrent T2DM in the PBC patients.

**Figure 2:**
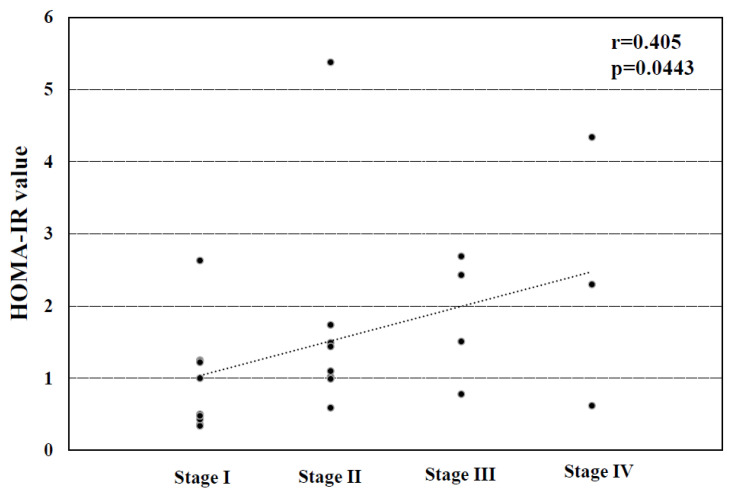
The correlation between the HOMA-IR values and the histological stages in the PBC patients.

We next investigated the correlation between the HOMA-IR value and the histological stage in the PBC patients. As indicated in Figure 2, the patients’ HOMA-IR values were significantly associated with their histological stages (r = 0.405, *p* = 0.0443).

### 3.5. Analyses of the Factors Affecting Serum AIM Levels in the PBC Patients

We explored which factors were significantly correlated with serum AIM levels in the PBC patients. As shown in Table 4, the BMI and HOMA-IR values were not associated with serum AIM levels in those patients, suggesting that the serum AIM level was independent of obesity and insulin resistance. However, the histological stage (r = 0.414, *p* = 0.0165), serum IgG level (r = 0.5329, *p* = 0.0012), and TNF-α level (r = 0.4224, *p* = 0.0130) were significantly correlated with the serum AIM level and inversely correlated with the serum Alb level (r = −0.4962, *p* = 0.0028) in the PBC patients, implying that the serum AIM levels were associated with the disease severity. Unfortunately, the multivariate analysis did not elucidate the significant factors that were correlated with the serum AIM levels (Table 5). No significant correlation was observed between the serum AIM levels and the titers of autoantibodies tested in this study or the serum IgM levels.

## 4. Discussion

We observed that the prevalence of obesity in this study’s 48 patients with PBC was approximately the same as those in patients with other autoimmune diseases [10]. The results of our analyses also confirmed that obesity was significantly associated with hepatic steatosis in PBC patients, which was almost similar to a previous finding [24], although the prevalence of hepatic steatosis was not as common as that reported [25]. This may be attributed to the difference in the severity of obesity among the study populations. The association between obesity and hepatic steatosis had already been observed in patients with MASLD [26] or HCV-related CLD [27].

Notably, the present study’s obese PBC patients had significantly lower serum ALP levels compared to the PBC patients without obesity. This novel finding may be derived from the inhibition of the intestinal type of ALP synthesis due to obesity [28]. The measurement of the ALP isozyme would enable us to confirm the speculation. Unfortunately, our analyses did not reveal sufficient evidence regarding whether the serum AIM was associated with the severity of obesity in patients with PBC. Contrary to the findings obtained in previous studies [5,6], obesity was not associated with disease severity in our PBC patients for as-yet unknown reasons.

Our findings demonstrated that the prevalence of concurrent T2DM in PBC was approximately equivalent to that in SLE [29], and that concurrent T2DM affected the disease severity in PBC patients, as has been reported in patients with MASLD or HCV-related CLD [1,8,9]. We also observed that insulin resistance was significantly correlated with the PBC patients’ histological stage, as has been demonstrated in HCV-related CLD [30]. However, our investigation did not elucidate the correlation between the serum AIM levels and the HOMA-IR values in patients with PBC.

It is also important that our PBC patients’ serum AIM levels were significantly related to their histological stages. A close correlation between the serum AIM levels and the degrees of liver fibrosis was observed in patients with HCV-related CLD [31,32]. We thus suspected that AIM may also be involved in the progression to hepatic fibrosis in PBC patients. However, the present PBC patients’ serum AIM levels were not correlated with their titers of anti-gp210, which reflects advanced liver damage [33]. This discrepancy seems theoretically paradoxical. The mechanism by which AIM is involved in liver fibrosis remains uncertain at present. AIM may participate in liver fibrosis via several factors other than insulin resistance in patients with liver fibrosis. To the best of our knowledge, the molecule which AIM may affect in liver fibrosis has not been identified yet. Further research is necessary to clarify the contribution of AIM to liver fibrosis in patients with PBC.

We also observed that the serum IgG levels were associated with the serum AIM levels in the PBC patients, although the titers of anti-M2, anti-gp210, and anti-Sp100; the positive rate of ACA; and the serum IgM level were not correlated. Serum AIM levels were also significantly elevated in the present AIH patients compared to those in the HCs. These results may imply that AIM was partially involved in the autoimmune response in the PBC patients, although a putative mechanism was not in the category for an obesity-induced autoimmune response [17]. The elevation of serum AIM levels was also confirmed in patients with SLE [34].

There are several limitations of the present study to consider. This study was a retrospective cohort investigation conducted with patients recruited from a single center, and the sample size was thus limited. The ages in the HC group did not match those in the PBC group, although the ages in the AIH group were approximately the same as those in the PBC group. Moreover, autoantibody levels and HOMA-IR values were not obtained from several patients. Multi-center clinical trials should be conducted to verify our findings. However, several valuable results were obtained from this study in spite of the small size sample, including the influence of concurrent T2DM in PBC patients and the involvement of AIM in the disease severity of PBC. Secondly, the study population included several patients who had been taking anti-diabetic medicines. The patients who took antidiabetic medicines were included in the statistical analyses. This may account for the comparatively favorable blood sugar control in the PBC patients with concurrent T2DM. Third, we did not conduct longitudinal analyses, and we thus could not confirm longitudinal changes in serum AIM levels. In addition, the correlations between serum AIM levels and serological hallmarks for liver fibrosis were not investigated in the patients with PBC.

## 5. Conclusions

In summary, the serum AIM levels of patients with PBC were not associated with obesity or insulin resistance, but were associated with the patients’ disease severity. These results may indicate that AIM is involved in the process of liver fibrosis in PBC patients. Further clinical trials to elucidate the involvement of AIM in the disease severity are desired, including the evaluation of liver fibrosis and the response to ursodeoxycholic acid (UDCA).

## Figures and Tables

**Table 1:** The clinical characteristics of patients with PBC.

Age (y.o.)	59.6 ± 11.2 (19–78)
Gender (male/female)	3/45
BMI	22.1 ± 2.9 (18.0–28.9)
HOMA-IR value	1.51 ± 1.23 (0.34–5.38)
Concurrent T2DM	7 (14.6%)
Scheuer’s classification(I/II/III/IV)	17/16/8/4
Hepatic steatosis (grade 1/grade2/grade3)	5/0/0

**Table 2:** Comparisons of clinical parameters between the PBC patients with and without concurrent obesity.

	Obesity (n = 12)	Non-Obesity (n = 36)	*p*-Value
Gender (Male/Female)	1/11	2/34	0.9999
Age (y.o.)	61.6 ± 7.5	59.0 ± 12.2	0.5035
BMI	26.1 ± 1.1	21.1 ± 2.1	<0.0001
Scheuer’s classification	2.1 ± 0.6 (n = 9)	1.9 ± 1.0 (n = 36)	0.6484
Frequency of steatosis (%)	4 (40.0%) (n = 10)	1 (2.9%) (n = 35)	0.0062
T-Bil (mg/dL)	0.8 ± 0.8	0.9 ± 1.0	0.7086
ALP (IU/L)	383 ± 126	650 ± 383	0.0172
ALT (IU/L)	40 ± 28	53 ± 26	0.1891
Alb (mg/dL)	3.9 ± 0.6	3.9 ± 0.6	0.7803
T-Cho (mg/dL)	215 ± 36 (n = 10)	207 ± 36 (n = 27)	0.5334
TG (mg/dL)	160 ± 128 (n = 9)	115 ± 84 (n = 24)	0.2451
IgM (mg/dL)	445 ± 260	475 ± 400	0.8134
IgG (mg/dL)	1857 ± 423	1699 ± 568	0.1633
anti-M2 (Index)	117.8 ± 68.0	89.4 ± 78.3	0.2838
anti-gp210 (Unit)	44.0 ± 44.1 (n = 7)	27.9 ± 43.0 (n = 27)	0.3879
anti-Sp100 (Unit)	27.4 ± 42.9 (n = 5)	9.5 ± 16.9 (n = 28)	0.3145
Positive rate for ACAs (%)	12.5 (n = 8)	27.3 (n = 33)	0.3578
HOMA-IR value	2.2 ± 1.6 (n = 7)	1.3 ± 1.0 (n = 18)	0.1085
HbA1c (%)	5.7 ± 0.4 (n = 4)	5.6 ± 0.9 (n = 13)	0.9023
AIM (ng/mL)	36.6 ± 33.2 (n = 7)	32.2 ± 28.3 (n = 27)	0.7233

**Table 3:** Comparisons of clinical parameters between the PBC patients with and without concurrent T2DM.

	Concurrent T2DM (n = 7)	Non-Concurrent T2DM (n = 41)	*p*-Value
Gender (Male/Female)	1/6	2/39	0.9159
Age (y.o.)	64.5 ± 3.8	58.9 ± 11.8	0.2607
BMI	21.9 ± 2.2	22.3 ± 3.0	0.4500
Scheuer’s classification	2.8 ± 1.0 (n = 6)	1.8 ± 0.9 (n = 39)	0.0313
Frequency of steatosis (%)	2 (33.3%) (n = 6)	3 (7.7%) (n = 39)	0.1248
T-Bil (mg/dL)	0.7 ± 0.2	0.9 ± 1.1	0.5928
ALP (IU/L)	642 ± 293	573 ± 368	0.6428
ALT (IU/L)	68 ± 24	47 ± 27	0.0486
Alb (mg/dL)	3.8 ± 0.3	3.9 ± 0.6	0.7397
T-Cho (mg/dL)	196 ± 24 (n = 6)	211 ± 37 (n = 31)	0.3386
TG (mg/dL)	105 ± 36 (n = 5)	131 ± 105 (n = 28)	0.2451
IgM (mg/dL)	597 ± 357	446 ± 373	0.3237
IgG (mg/dL)	1779 ± 432	1728 ± 558	0.8192
anti-M2 (Index)	71.7 ± 58.7	100.0 ± 78.7	0.3686
anti-gp210 (Unit)	40.6 ± 51.9 (n = 5)	29.6 ± 42.2 (n = 29)	0.6069
anti-Sp100 (Unit)	5.3 ± 2.3	14.7 ± 26.9	0.4480
Positive rate for ACAs (%)	20.0 (n = 5)	25.0 (n = 36)	0.6466
HOMA-IR value	2.6 ± 1.8 (n = 3)	1.4 ± 1.1 (n = 22)	0.1085
HbA1c (%)	6.4 ± 0.5 (n = 6)	5.2 ± 0.7 (n = 11)	0.0030
AIM (ng/mL)	38.7 ± 40.3 (n = 5)	32.1 ± 27.3 (n = 29)	0.6453

**Table 4:** Analyses of factors associated with serum AIM level in the patients with PBC.

Parameter	Correlation Coefficient	*p*-Value
BMI	0.1766	0.3178
Scheuer’s classification	0.4144	0.0165
T-Bil	0.328	0.0582
ALP	−0.4962	0.4783
ALT	−0.0075	0.9663
Alb	−0.4962	0.0028
T-Cho	−0.2892	0.1435
TG	0.005	0.9809
IgM	0.0781	0.6607
IgG	0.5329	0.0012
anti-M2	0.1322	0.4561
anti-gp210	0.0964	0.5876
anti-Sp100	−0.0312	0.8632
ACAs	−0.0124	0.9446
HOMA-IR	0.1608	0.4746
TNF-a	0.4224	0.0130

**Table 5:** Multivariate regression analysis for factors associated with serum AIM levels in the patients with PBC.

Variable	95% Confidential Interval	*p*-Value
Scheuer’s classification	−11.7089–13.7129	0.8730
Alb	−31.7191–3.1765	0.1051
IgG	−0.0019–0.0402	0.0733
TNF-a	−0.0934–1.7735	0.0759

## Data Availability

The data presented in this article are available from the corresponding author upon reasonable request.

## Abbreviations

ACA	Anti-centromere antibody
AIH	Autoimmune hepatitis
AIM	Apoptosis inhibitor of macrophage
Alb	Albumin
ALP	Alkaline phosphatase
ALT	Alanine aminotransferase
AMA	Antimitochondrial antibody
BMI	Body mass index
CLD	Chronic liver disease
HbA1c	Hemoglobin A1c
HC	Healthy control
HCV	Hepatitis C virus
HOMA-IR	Homeostasis model for the assessment of insulin resistance
IgM	Immunoglobulin M
MASLD	Metabolic dysfunction-associated steatotic liver disease
PBC	Primary biliary cholangitis
SLE	Systemic lupus erythematosus
T-Cho	Total cholesterol
T2DM	Type 2 diabetes mellitus
TNF-α	Tumor necrosis factor-α
T-Bil	Total bilirubin
TG	Triglyceride
